# MiR-615 Agomir Encapsulated in Pluronic F-127 Alleviates Neuron Damage and Facilitates Function Recovery After Brachial Plexus Avulsion

**DOI:** 10.1007/s12031-021-01916-5

**Published:** 2021-09-26

**Authors:** Kangzhen Chen, Lu Ding, Hua Shui, Yinru Liang, Xiaomin Zhang, Tao Wang, Linke Li, Shuxian Liu, Hongfu Wu

**Affiliations:** 1 Department of Anesthesiology, Guangzhou Huadu Affiliated Hospital of Guangdong Medical University (Guangzhou Huadu District Maternal and Child Health Care Hospital), Guangzhou, 510800 China; 2 Key Laboratory of Stem Cell and Regenerative Tissue Engineering, Guangdong Medical University, Dongguan, 523808 China; 3 Scientific Research Center, the Seventh Affiliated Hospital, Sun Yat-sen University, Shenzhen, 518107 China; 4 Department of Surgery, The Third Hospital of Guangdong Medical University (Longjiang Hospital of Shunde District), Foshan, 528318 China

**Keywords:** MiR-615, Pluronic F-127(PF-127), Motoneuron, Brachial plexus avulsion (BPA)

## Abstract

Brachial plexus avulsion (BPA) is a devastating traumatic peripheral nerve injury complicated with paralysis of the upper extremity. We previously reported that leucine-rich repeat and immunoglobulin-like domain-containing NOGO receptor-interacting protein 1 (LINGO-1) has a potent role in inhibiting neuron survival and axonal regeneration after the central nervous system (CNS) damage and miR-615 is a potential microRNA (miRNA) negatively regulated LINGO-1. However, the effect of miR-615 in BPA remains to be elucidated. Accumulating evidence indicates that pluronic F-127 (PF-127) hydrogel could serve as a promising vehicle for miRNA encapsulation. Thus, to further explore the potential role of hydrogel-miR-615 in BPA-reimplantation, the present study established the BPA rat model and injected miR-615 agomir encapsulated by PF-127 hydrogel into the reimplantation site using a microsyringe. In this study, results indicated that hydrogel-miR-615 agomir effectively alleviated motoneuron loss by LINGO-1 inhibition, promoted musculocutaneous nerve regeneration and myelination, reduced astrocytes activation, promoted angiogenesis and attenuated peripheral amyotrophy, leading to improved motor functional rehabilitation of the upper extremity. In conclusion, our findings demonstrate that miR-615-loaded PF-127 hydrogel may represent a novel therapeutic strategy for BPA treatment.

## Introduction

Brachial plexus avulsion (BPA) involves injury of the C5-T1 spinal nerves, which is intimately responsible for cutaneous and muscular innervation of the upper extremity. Injury to the brachial plexus can occur as a result of motorcycle accidents, obstetric trauma (Smith et al. 2018), tumors, and/or inflammation (Chen et al. 2017), leading to massive motoneuron death, axonal damage, amyotrophy and severe functional deficits of the upper extremity. After peripheral nerve injury, motoneuron survival is an essential prerequisite of neurofunctional recovery. Although general supportive treatment (Wu et al. 2003), nerve transposition repair (Midha and Grochmal 2019), and stem cell transplantation (Araujo et al. 2017) exert multiple beneficial neuronal effects for the BPA treatment, the limited curative effect has been shown in clinical practice. Therefore, an effective therapeutic strategy that alleviates neuronal death and accelerates surviving neurons regeneration is vital and worthy for BPA treatment.

LINGO-1 is a cell-surface glycoprotein selectively expressed on neurons and oligodendrocytes in CNS (Zhang et al. 2009). It is reported that LINGO-1 could negatively regulate neuron and oligodendrocyte survival, neurite extension, axon regeneration and axon myelination,  and inhibit neural stem cells and oligodendrocyte precursor cells differentiation (Mi et al. 2008). Our previous work has shown that transplantation of LINGO-1 shRNA loaded by pluronic F-127 could improve the function recovery after BPA through protecting motoneurons survival, promoting axonal regeneration and facilitating neovascularization (Ding et al. 2019). Despite those evidences that LINGO-1 was involved in the pathogenesis of CNS injury, the potential regulators of LINGO-1 have rarely been studied. Importantly, our recent study has identified miR-615 as a potential miRNA that directly targets LINGO-1 (Wu et al. 2020).

MicroRNAs, endogenous small noncoding RNAs, could silence gene expression through binding the 3′-UTR of target mRNA, leading to protein degradation or translation repression (Bartel 2009). Numerous studies have shown that miRs play vital roles in neurological disorders. MiR-615 is an intronic miRNAs, and its host gene is HoxC5, a member of Hox protein family. It has been proved that Hox protein is a pivotal determinant of spinal motoneuron identity and organization (Dasen et al. 2003; Woltering and Durston 2008). Moreover, the expression of miR-615 was obviously upregulated at the differentiated embryonic stem cells, neural stem cells, and neuroblastoma cell lines (Stallings et al. 2011; Tripathi et al. 2011), implicating that miR-615 might be involved in the neuronal fate determination. Importantly, our previous researches have revealed that miR-615, by targeted suppression of LINGO-1, facilitated the proliferation and neuronal differentiation of neural stem cells. Besides, miR-615 also promoted axonal regeneration and remyelination as well as function recovery of SCI rats, indicating that miR-615 might be a promising therapeutic target for nervous system injury (Wu et al. 2020). However, it is unknown whether miR-615 could also exert neuroprotective role after BPA, which induces the most serious neuronal loss and death.

Pluronic F-127 (PF-127), a synthetic non-cytotoxic hydrogel, is approved by the US Food and Drug Administration (FDA) for use in humans (Diniz et al. 2015). A promising characteristic of PF-127 is its thermosensitivity, which makes it useful for encapsulating releasing therapeutics after injection into tissues. PF-127 have been explored widely for injection into CNS tissue (Wu et al. 2013; Ding et al. 2019; Wang et al. 2019) and have been identified as safe in most animals. Previous studies showed that PF-127 could transfer LINGO-1 shRNA to the brachial plexus avulsion site, leading to improved levels of functional recovery (Wu et al. 2013; Ding et al. 2019; Wang et al. 2019). Additionally, in a spinal cord trauma model, hirudin encapsulated in PF-127 promotes functional recovery from a demyelination lesion (Sellers et al. 2014). Therefore, PF-127 is a stable, biocompatible, and injectable nontoxic hydrogel that is suitable to delivery therapeutics. Based on the advantage of PF-127, using PF-127 hydrogel with embedded miR-615 agomir, we sought to prolong delivery of miR-615 agomir at the avulsion site.

In this study, we identified miR-615 as a novel neuronal protector that plays a critical role in preserving damaged neurons by inhibiting LINGO-1 in BPA rats. In addition, co-grafting of miR-615 agomir with PF-127 hydrogel improved motor function recovery follow BPA reimplantation by alleviating neuron damage, promoting nerve fiber regeneration, relieving the astrocyte activation, and attenuating amyotrophy. Taken together, this research provides evidence that miR-615 functions as a negative regulator of LINGO-1 and is a potential therapeutic target for BPA repair.

## Materials and Methods

### MiR-615 Agomir and Hydrogel Preparation

To obtain higher biostability in animal experiments, miR-615 agomir and its negative control-agomir (NC-agomir) were synthesized through a particular chemical modification process. The miR-615 agomir and its negative control were supported by RiboBio (Guangzhou, China).

Pluronic F-127 (Sigma, Aldrich, USA) was prepared as follows: PF-127 hydrogel powder was mixed with 0.1 M phosphate-buffered saline (PBS, pH = 7.6), and a 25% (w/v) suspension was obtained. The mixture was shaken gently at 4 ℃ overnight to fully dissolve into solution and stored at 4 ℃ for further use after filtrating with a filter (0.22 µm). All procedures were carried out under aseptic conditions.

### Establishment of BPA-Reimplantation Rat Model

Seventy-five adult female Sprague–Dawley (SD) rats (age, 8–10 weeks; weight, 180–220 g) supplied by the Experimental Animal Center of Southern Medical University (Guangdong, China) were used in this study. All procedures using laboratory animals were conducted in compliance with the Guide for the Care and Use of Laboratory Animals (National Research Council 1996) and approved by the Administration Committee of Experimental Animals, Guangdong Province, China.

Before the operation, all rats were anesthetized with 1% pentobarbital sodium (40 mg/kg) intraperitoneally. After skin preparation, an incision along the centerline of the animal body in the skin and muscles was prepared, and the spinal segments from the 4th cervical (C4) to 2nd thoracic (T2) lamina were exposed. Subsequently, a unilateral dorsal laminectomy of the right C5 to C7 laminae was performed to expose the dorsal root. Microscissors were used to remove the right C5–C7 dorsal roots, and the corresponding ventral roots were avulsed using a slender glass hook under the stereomicroscope. The C5 and C7 spinal nerves were cut partly, leaving an obvious gap between the nerve roots and spinal cord. The C6 ventral root was replanted to the avulsed site for regeneration. The Terzis grooming test was performed for the upper extremity on the right side on the first day after surgery. All animals scoring 0 indicated the successful establishment of BPA-reimplantation models

### Transplantation of MiR-615 Agomir and PF-127 Hydrogel Into BPA Rats

In order to determine the role of miR-615 in vivo, the BPA rats were randomly divided into five groups: PBS group (*n* = 15), NC-agomir group (*n* = 15), miR-615 agomir group (*n* = 15), miR-615 agomir + gel group (*n* = 15), and gel group (*n* = 15). After establishment of BPA model, each group received respective implants (10 µL PBS, 10 µL NC-agomir, 5 µL miR-615 agomir+5 µL PBS, 5 µL miR-615 agomir+5 µL gel, 10 µL gel) in the avulsion site of the C6 ventral root through 10 µL Hamilton syringe. The needle remained in reimplanted site for 2 min and then slowly removed. After transplantation, the wound was disinfected and closed. The animals were placed on an electric blanket until emergence from anesthesia. All rats were intraperitoneally administrated with penicillin streptomycin (10,000 U/mL; Thermo Fisher Scientific, Waltham, MA, USA) once a day during the first-week post-operation to prevent infection.

### Gross Specimen Analysis

At the end of 6-week survival period, rats were perfused after overdose anesthesia, and both sides of C5–7 cervical spinal cord, biceps, and coterminous musculocutaneous nerves were carefully separated. The gross specimen was fixed in 4% paraformaldehyde at 4 ℃ for further observation.

### Hematoxylin and Eosin Staining

Bicep paraffin sections were collected at 6 weeks post-operation to perform hematoxylin and eosin (H&E) staining. The sections were dewaxed in xylene, dehydrated by graded ethanol, and rinsed with distilled water. Subsequently, the bicep sections were stained with hematoxylin solution and counterstained with eosin solution for 30–60 s after differentiation in 1% acid alcohol for 30 s and soak in running water. After rinsing with distilled water for 5 min, the sections were again dehydrated by graded ethanol and cleared by xylene. Finally, the sections were mounted onto coverslips with natural resin. More than six fields of each bicep were randomly photographed using a light microscope (20×, ECLIPSE TS100; Nikon, Tokyo, Japan). The muscle fiber diameter was measured and quantified using ImageJ software. The extent of fibrosis was determined by the ratio of fibroblast nuclei number in ipsilateral to contralateral biceps.

### Nissl Staining

To observe the neurons survival in the C6 spinal segments, cross-sections of the spinal cord were collected and performed Nissl staining at 6 weeks post-surgery. Sections were washed in PBS three times, and 5 min each time. After dehydration in graded ethanol, samples were stained in 0.05% toluidine blue for 30 min and washed in distilled water three times (1 min each time). Subsequently, C6 spinal cord sections were differentiated in 95% alcohol for 10 min, dehydration in absolute ethanol (I, II; 1 min each), cleared in xylene (I, II; 5 min each), and mounted with neutral resins.

### Western Blot Assay

At 1 week after surgery, the C6 spinal cord segments were quickly dissected. Protein from the C6 segments was extracted using protein lysis buffer. Equal amounts of total protein were electrophoresed on 10% SDS-PAGE, transferred onto polyvinylidene fluoride (PVDF) membrane (0.2 µm; Millipore), and then blocked with 5% defatted milk in TBS with 0.05% Tween (TBST) for 2 h at room temperature. The membranes were incubated with rabbit anti-LINGO-1 antibody (1:1000, Sigma, USA), NeuN (1:1000; Millipore, USA), and glyceraldehyde 3-phosphate dehydrogenase (GAPDH) (1:1000; Thermo Fisher, USA) overnight at 4 ℃. After washing in TBST, three times for 5 min each, primary antibodies were detected with secondary antibodies as follows for 2 h at room temperature: horseradish peroxidase goat anti-mouse/rabbit IgG (1:5000; ABclonal, USA). Blots were then washed as described above and visualized by chemiluminescence (ECL) kit (Pierce, USA). The density of the immunoreactive bands was analyzed using ImageJ software. GAPDH was used as the internal control.

### Behavioral Test

The motor function of the upper extremity was evaluated by Terzis grooming test (TGT) weekly. The entire test process was conducted in a spacious and quiet environment. Using a 50-mL syringe to spray bacterial-free water on the neck of rats to elicit grooming behavior of the bilateral upper extremities. The function of the right upper extremity was assessed by the following 0–5 point scale: grade 0, the upper extremity of affected side does not respond; grade 1, the elbow of affected side can bend, but the upper extremity of affected side cannot touch nose; grade 2, the upper extremity of affected side can touch nose; grade 3, the elbow of affected side can bend and the forelimb of affected side can touch the site below the eyes; grade 4, the forelimb of affected side can touch the eyes; grade 5, the forelimb of the affected side can touch the ears or back of the ears.

Before operation, all animals were evaluated and scored 5. Twenty-four hours after operation, all animals were reevaluated and exhibited a successful grade 0. From the first week post-operation to the endpoint, the Terzis grooming test was performed weekly and recorded by two observers who were blinded to group assignment. If there exists a disagreement, the test was assessed by the third people.

### Fluorogold Retrograde Tracing

To observe the spinal motor neurons, the fluorogold retrograde labeling was performed at 2 days before the 6-week endpoint. Animals from each group were anesthetized by intraperitoneal injection and preoperative skin preparation was performed. Then, their right musculocutaneous nerve was exposed under stereoscopic microscopy, and a total of 0.5 µL fluorogold (Sigma–Aldrich) was slowly injected into using a micropump at 4.0 ± 0.5 mm distal to the avulsed site of the lateral cord. At 2 days after injection, animals were perfused and C5–C7 spinal cord segments were collected. Spinal cord segments were fixed in 4% paraformaldehyde overnight and dehydrated in 30% sucrose for 2 days. Subsequently, 20-µm frozen sections of the spinal segments were collected. Fluorogold-retrograde-labeled motor neurons of C5–7 spinal cord ventral horn in affected side were calculated under fluorescence microscopy (Carl–Zeiss Axioplan 2 imaging E, Baden–Wurttemberg, Germany).

### Electron Microscopy Observation

In order to observe the axonal structure of musculocutaneous nerve in each group, rats were perfused with 0.9% normal saline followed by a mixture of 4% PFA and 25% glutaraldehyde (*v*/*v* = 9:1), and the right musculocutaneous nerve was dissected and extracted for electron microscopy at 6 weeks post-surgery. The right musculocutaneous nerve was fixed in 4% glutaraldehyde (SPI-CHEM, USA) for 4 h at 4 ℃ and washed three times with 0.1 M sodium cacodylate buffer. Subsequently, the tissues were fixed in 1% osmium tetroxide for at least 1 h and washed three times with distilled water. After that, the tissues underwent gradient ethanol hydration to dehydration and were embedded with epoxy resin. The tissues were then heated to 60 ℃ for 48 h and cut into 90-nm ultrathin cross-section. Finally, the sections were stained with 2% uranyl acetate and lead citrate and examined using a transmission electron microscope.

### Electrophysiology

To explore whether the motor functional recovery is related to the electromyography changes of the forelimbs, electrophysiology was performed. Firstly, the right biceps brachii and homolateral musculocutaneous nerve were dissected, then the stimulating electrode was hooked in musculocutaneous nerve, while recording electrodes were inserted into the biceps with the depth of 1–2 mm and distance of 3–9 mm. Electrode stimulation intensity was 0.8–1.2 V, and electrical activity was performed at three different motor unit locations in each bicep. Stimulation was delivered by the same electrical stimulator from MedLab Biological Signal Collection System (Meiyi Technology Ltd., Nanjing, China).

### Immunofluorescence Staining

To observe the condition of neuronal survival, astrocytes activation, and angiogenesis, C6 spinal segments in BPA animals receiving different treatments were determined using immunohistochemistry staining. The primary antibodies used were the following: rabbit polyclonal anti-NeuN (neuronal marker; 1:200; Millipore), rabbit polyclonal anti-glial fibrillary acidic protein (GFAP) (astrocyte activation marker; 1:200; Thermo Fisher), and mouse polyclonal anti-CD31 (angiogenesis marker; 1:200; Millipore). The secondary antibody used was mouse anti-rabbit conjugated to Alexa Fluor 568 or goat anti-rat conjugated to Alexa Fluor488 (1:400, Thermo Fisher Scientific). Briefly, after permeabilization in 0.3% Triton X-100 for 15 min, the sections were blocked in 10% natural goat serum for 30 min. Then, sections were incubated with primary antibodies overnight at 4 °C. The next day, sections were incubated with secondary antibody in the dark for 2 h at 37 °C after washing three times in PBS. After repeated washes, sections were counterstained with 4′,6-diamidino-2-phenylindole (DAPI) (1:5000, Cell Signaling Technology) for 15 min. Finally, a fluorescence mounting medium (Dako, Copenhagen, Denmark) was used to mount tissue onto coverslips.

### Statistical Analysis

The data were analyzed using one-way analysis of variance (ANOVA) or two-way analysis of variance using SPSS (version 20.0). All data were presented as means ± standard deviation (SD). *P* < 0.05 was considered statistically significant.

## Results

### MiR-615 Agomir+Gel Treatment Alleviated Muscle Atrophy after BPA- Reimplantation

**Fig. 1:**
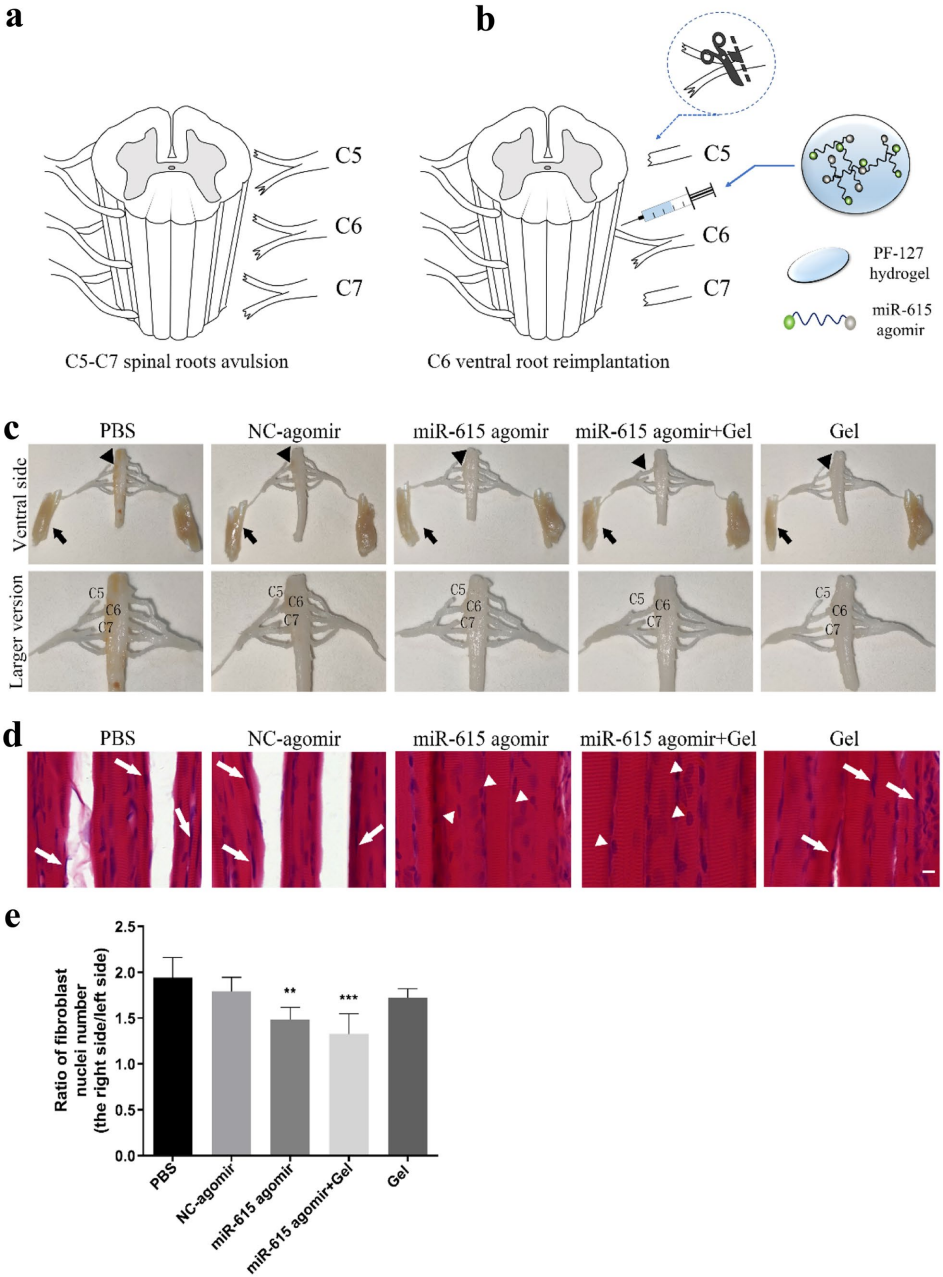
Co-transplantation of miR-615 agomir with gel alleviated muscle atrophy after avulsion/reimplantation. **a** Right-sided avulsion of C5–C7 spinal nerve roots and reimplantation of C6 ventral root into the surface of the corresponding spinal segment. **b** The injector indicates the reimplanted site and the miR-615 agomir and pluronic F-127 hydrogel injection site. **c** Representative pictures of gross anatomical specimen of rats with BPA reimplantation from different groups. Reimplanted C6 ventral roots (black triangle) tightly connected to the original avulsion site. On the contrary, two obvious defects were observed between C5 and C7 nerve roots and the corresponding spinal segments. Compared with the healthy side, atrophied biceps were common in the affected side (black arrows). **d** Longitudinal sections of the right affected biceps by hematoxylin and eosin staining. In PBS and NC-agomir groups, shrunken sarcoplasm and large fibroblast nucleus (white arrow) were common in atrophied biceps. Instead, miR-615 agomir and miR-615 agomir+gel group showed less fibrosis, demonstrated by fewer fibroblast nucleus and a great many of clear myocyte nuclei (white triangle). **e** The level of fibrosis was decided by the ratio of fibroblast nuclei number in ipsilateral to contralateral biceps. Data are presented as the mean ± SD (one-way analysis of variance followed by the least significant difference post hoc test). **p < 0.01, ***p < 0.001, vs. PBS group. Scale bar (C): 100 μm

To investigate the possible role of miR-615 after brachial plexus injuries, BPA-reimplantation surgery was conducted on rats (Fig. 1a), and miR-615 agomir, with or without the PF-127 hydrogel, was immediately injected into the avulsed site (Fig. 1b).

After dissection of injured animals at six-week post-BPA, all replanted C6 ventral roots were found to be successfully connected with the surface of the corresponding spinal segment in each group, while an obvious laceration was observed in C5 and C7 roots. Mild inflammation or fibrotic reaction was detected at the reimplant site. The reimplanted C6 ventral roots were similar to the counterparts of the health side in dimensions and length. However, remarkable nerve root retraction was observed in avulsed C5/C7 ventral roots. Moreover, the biceps of affected side exhibited obvious atrophy compared with the contralateral ones, which mainly results from the denervation of musculocutaneous nerve (Fig. 1c).

Denervated muscle not only leads to muscle atrophy and fibrosis but also motor dysfunction. To evaluate the pathological changes of biceps brachii atrophy after surgery, the biceps brachii muscles of both the ipsilateral and contralateral forelimbs were assessed by H&E staining. For the sake of assessing the extent of muscle fibrosis, the ratio of fibroblast nuclei in the ipsilateral to the contralateral biceps was calculated. As illustrated in Fig. 1d, e, muscle fibers in PBS group displayed much smaller diameters, unclear myocyte, obvious fibrosis, and partial rupture, suggesting apparent muscle atrophy of the avulsed biceps. Conversely, when treated with miR-615 agomir and miR-615 agomir+gel, muscle fibers with larger diameters, clearer myocyte, and less fibroblasts were observed, indicating significant therapeutic effects of miR-615 agomir with or without gel on muscle atrophy. There is evidence suggestive of a beneficial role of gel on hemostasis, biocompatibility, and biodegradation, although the effect of gel alone in this study on preventing biceps brachii atrophy was no significant. Taken together, these findings demonstrated that transplantation of miR-615 agomir or miR-615 agomir with gel effectively alleviated biceps brachii atrophy after BPA-reimplantation, especially in the latter group.

### MiR-615 Agomir+Gel Treatment Facilitated the Survival of Motor Neurons After Avulsion/Reimplantation

**Fig. 2:**
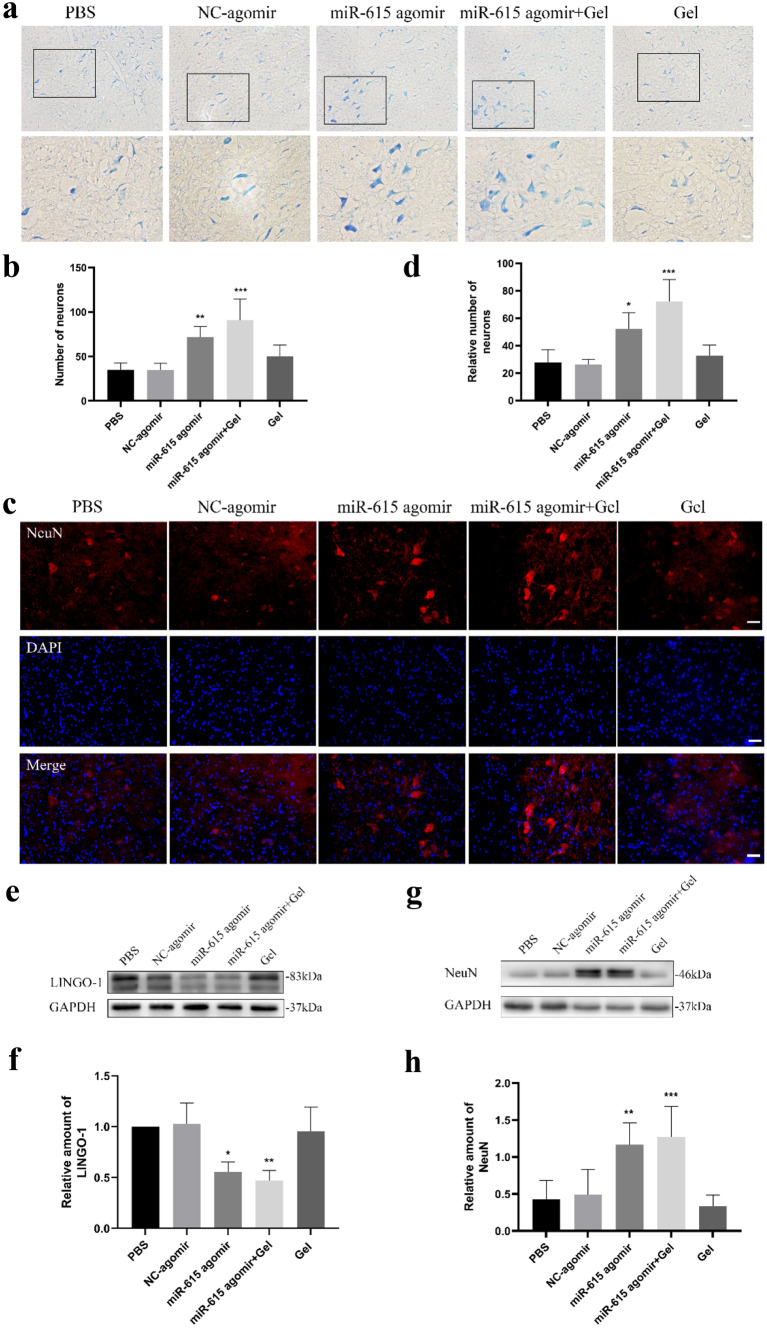
Co-transplantation of miR-615 agomir with gel stimulated axonal regeneration and reduced neuronal death in affected spinal segments after avulsion/reimplantation. **a, b** Representative micrographs and quantitative analysis of the survival neurons around the avulsed epicenter by Nissl’s staining at low and high magnification, respectively, indicating a significant increasement of neurons in miR-615 agomir+gel rats. **c** Immunofluorescence photos of neurons around the avulsed site in the ipsilateral C6 segment. **d** Relative number of neurons were measured and counted. **e, f** Western blot assay and relative quantification of LINGO-1 protein demonstrated dramatic downregulation of LINGO-1 in miR-615 agomir and miR-615 agomir+gel group. The results illustrated that miR-615 negatively regulated LINGO-1. GAPDH was used as an internal control. **g, h** Western blot assay and relative quantification of NeuN, a neuron-specific protein, demonstrated dramatic upregulation of NeuN in miR-615 agomir and miR-615 agomir+gel group. GAPDH was used as an internal control. Data are presented as the mean ± SD (one-way analysis of variance followed by the least significant difference post hoc test). *p < 0.05, **p < 0.01, ***p < 0.001, vs. PBS group. Scale bar (a—upper row) = 500 μm, scale bar (b—lower row) = 250 μm, Scale bar (c) = 200 μm

Spinal root avulsion usually leads to motoneuron degeneration and death in the corresponding spinal cord. The surviving neurons of avulsed C5–C7 spinal segments were detected by Nissl staining (Fig. 2a). Overall, the viable residual neurons in the miR-615 agomir and miR-615 agomir+gel groups were much more than those in the PBS group, especially in the latter (Fig. 2b). To assess neuronal injury and regeneration, we chose NeuN, a neuronal marker which is expressed in the nucleus of mature neurons. Consistently, immunofluorescence results show that the number of NeuN^+^ neurons was higher in groups treated with miR-615 agomir with gel than other groups (Fig. 2c, d), suggesting the protective role of miR-615 on motoneurons post-BPA.

To further explore the potential mechanism underlying miR-615 on neuron preservation, LINGO-1, a significant inhibitor in neuron survival and axonal regeneration was detected by western blot assay (Fig. 2e). The protein levels of LINGO-1 were significantly decreased in miR-615 and miR-615+gel groups (Fig. 2f), accompanied by the increased expression of NeuN (Fig. 2g, h), indicating that miR-615 might rescue motoneurons loss via LINGO-1 suppression. Besides, the PF-127 hydrogel did not disturb the integrity and bioactivity of the miR-615 agomir, for no significant difference was detected in miR-615 agomir and miR-615 agomir+gel groups.

### MiR-615 Agomir+Gel Treatment Was Beneficial to the Motor Functional Restoration of Monoplegia Right Upper Extremity

**Fig. 3:**
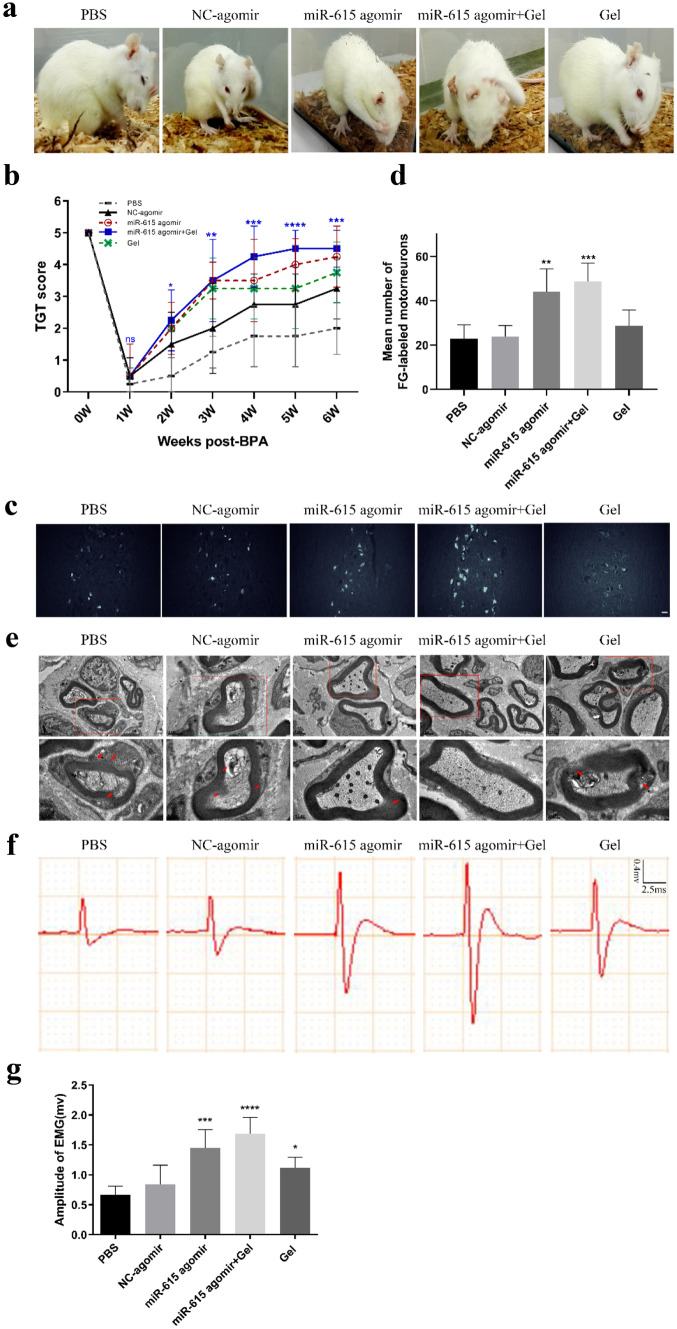
Co-transplantation of miR-615 agomir with gel improved the motor functional restoration of monoplegia right upper limb after avulsion/reimplantation. **a** Locomotion evaluation of animals in each group at sixth week after BPA-reimplantation. These pictures show a significant motor functional restoration that appeared on miR-615 agomir and miR-615 agomir+gel group, whereas the rats in other groups performed a little recovery. **b** Terzis grooming test was carried out weekly post BPA. Rats treated with miR-615 agomir+gel acquired the highest scores from the third week to sixth week after BPA reimplantation. **c** Fluorescence photomicrographs of FG-labeled motor neurons in ipsilateral C5–7 spinal segments. **d** Relative amounts of fluorogold-labeled motor neurons in ipsilateral C5–7 spinal cord. The number of fluorogold-labeled motor neurons in miR-615 agomir and miR-615 agomir+gel groups were more than PBS group. **e** Electron micrographs of musculocutaneous nerve. Distinct demyelination (red arrow) was observed in PBS, NC-agomir, and gel groups, yet the axons treated with miR-615 agomir with or without gel were more intact. **f** Electrophysiological activity of avulsed right forelimbs in each group at 6-week endpoint. **g** The amplitude of electromyography appears larger in the miR-615 agomir+gel group compared to PBS group, indicating better recovery of neuromuscular activity. Data are presented as the mean ± SD (one-way analysis of variance followed by the least significant difference post hoc test). *p < 0.05, **p < 0.01, ***p < 0.001, vs. PBS group. Scale bar (c) = 50 μm, Scale bar (e—upper row) = 1 μm, scale bar (e—lower row) = 0.5 μm

BPA usually causes the sensorimotor dysfunction of affected upper extremities. To investigate the effect of miR-615 agomir with gel on the motor functional recovery of the monoplegia right upper extremity after BPA, the Terzis grooming test was performed weekly in this study. Apart from paralysis in right upper extremity, all animals in each group barely exist obvious neurological defect after surgery. At the sixth week after BPA-reimplantation, most rats in miR-615 agomir and miR-615 agomir+gel groups could touch their eye or ear. By contrast, animals treated with PBS, NC-agomir, and gel hardly could touch their eye. (Fig. 3a) Within the first week after injury, all the animals displayed abnormal elbow flexion with a mean score of 0–1, indicating the motor function was almost completely lost and proved a successful operation. Within 1–3 weeks after injury, TGT scores increased from week to week in all surgical groups. Additionally, the mean TGT scores were dramatically improved from three to six weeks in miR-615 agomir and miR-615 agomir+gel groups after injury, when compared with PBS group. At the sixth week post-injury, the TGT score in two agomir-treated groups was the highest among other groups. Furthermore, rats in miR-615 agomir+gel treatment exhibited better locomotor performance and TGT scores than in miR-615 agomir alone (Fig. 3b).

To explore whether the improved motor functions were attributed to the axonal regeneration of motoneurons and injured brachial nerve, FG-retrograde tracing study was conducted at 2 days before perfusion (Fig. 3c). About 0.5 µL FG was injected into the musculocutaneous nerve at 4.0 ± 0.5 mm from the avulsion site. At the 6-week time point, only a few FG-labeled motoneurons were detected in the PBS group. However, we found a significant increase in the number of FG-positive motoneurons in rats treated with miR-615 agomir and miR-615 agomir+gel, which illustrated approximately 2-fold and 2.21-fold, respectively, in comparison to those in the PBS group (Fig. 3d). Collectively, these results indicated that the combination therapy could promote more regenerated motoneurons to extend their axons into the distal peripheral nerve trunk. Subsequently, the remyelination of distal musculocutaneous nerves was evaluated by electron microscope (EM) (Fig. 3e). After avulsion/reimplantation, electron micrographs revealed extensive demyelination in PBS, NC-agomir, and gel groups. In turn, the neuropathies in myelin sheath were dramatically attenuated in miR-615 agomir and miR-615 agomir+gel groups, signifying the improved myelination of eroded myelin sheath. Electrophysiology testing is critical for reflecting the status of muscles and nerve damage. Electrophysiology testing was performed to confirm the electrophysiological restoration of monoplegia right upper extremity (Fig. 3f). Compared with PBS group, the amplitudes of motor evoked potentials (MEPs) in the miR-615 agomir and miR-615 agomir+gel groups were higher. It was worth noting that the highest MEPs appeared in the miR-615 agomir+gel group. The mean MEPs detected in miR-615 agomir (1.4478 mv) and miR-615 agomir+gel groups (1.6836 mv) were approximately 2.16-time and 2.52-time in comparison to those in the PBS group (0.6676 mv), respectively (Fig. 3g). Together, these results indicated that miR-615 agomir and gel might promote injured nerves regeneration, myelination, and neural conduction functions, which was beneficial to the motor functional restoration of monoplegia right upper extremity.

### MiR-615 Agomir+Gel Treatment Improved Neovascularization and Reduced Astrocyte Activation after BPA Reimplantation

**Fig. 4:**
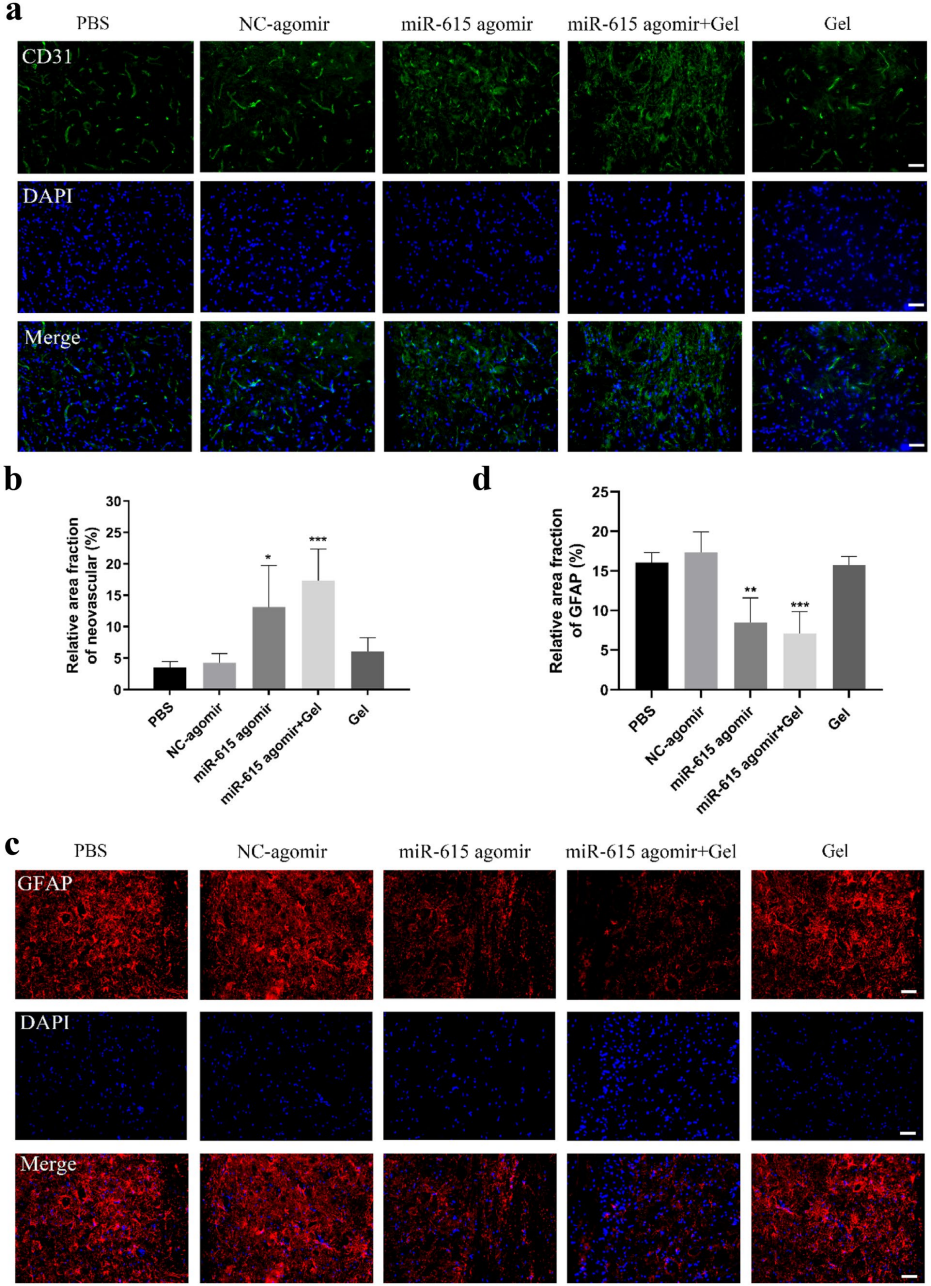
Co-transplantation of miR-615 agomir with gel enhanced neovascularization and reduced the astrocyte activation after brachial plexus avulsion. **a** Immunofluorescence photos of angiogenesis around avulsed site in the ipsilateral C6 segment. **b** Analysis of neurovascular areas at 6 weeks after surgery. The PBS rats exhibited few angiogeneses, while the rats in miR-615 agomir and miR-615 agomir+gel groups displayed a significant increase of neovascularization. **c** Immunofluorescence photos of GFAP around avulsed site in the ipsilateral C6 segment. **d** Relative area fraction of GFAP at 6 weeks after surgery. The miR-615 agomir and miR-615 agomir+gel groups rats exhibited few astrocyte activations, while the rats in PBS, NC-agomir, and gel groups displayed a significant increase of astrocyte activation. Data are presented as the mean ± SD (one-way analysis of variance followed by the least significant difference post hoc test). *p < 0.05, **p < 0.01, ***p < 0.001, vs. PBS group. Scale bar (a and c) = 200 μm

It is believed that the widespread vascular damage usually contributes to motoneuron death and functional recovery following brachial plexus avulsion. To analyze the revascularization after avulsion injury, an immunofluorescence assay of the neovascular endothelial cells marker CD31 in ipsilateral C6 spinal cord segments was conducted at six weeks post-injury (Fig. 4a). As shown in Fig. 4a, few new blood vessel formation in the PBS, NC-agomir, and gel groups were detected. On the contrary, miR-615 agomir and miR-615 agomir+gel administration significantly promoted angiogenesis, in which the relative CD31 staining areas were 3.61-fold and 5.00-fold compared with those in the PBS group, respectively (Fig. 4b). Taken together, these results suggested that miR-615 agomir and gel combination might improve the nerve regeneration through enhancing angiogenesis.

Excessive astrocyte activation is against for the axonal regeneration after BPA, which results in glial scar formation and many inhibitory molecules production. Reducing astrocyte scar is also one of the therapeutic strategies of SCI and BPA repair. In this study, we performed immunofluorescence using GFAP to evaluate the extent of astrocyte activation. As shown in Fig. 4c, astrocyte activation was significantly decreased by miR-615 agomir with or without gel compared with PBS application. Indeed, the relative area fraction of GFAP was approximately 2.27-time and 2.45-time mores in the miR-615 agomir+gel group compared with the PBS and NC-agomir groups (Fig. 4d). Taken together, co-graft of miR-615 agomir and gel might promote angiogenesis and reduce the level of astrocyte activation, which is beneficial to improve the axonal regeneration and motor function recovery.

## Discussion

BPA represents a sophisticated nerve injury in the upper extremity characterized by the sudden tear of rootlets from the surface of the spinal cord, accompanied by widespread spinal motoneuron degeneration and targeted muscle denervation (Yuan et al. 2000). BPA is responsible for the most serious injury of upper extremity and often occurs in young adults. Fortunately, various miRNAs have been involved in the neural system injury. The aim of current study is to evaluate the function of miR-615 in the BPA recovery. Results from the present study found that miR-615 agomir loaded by PF-127 hydrogel effectively rescued damage neurons, promoted the axonal regeneration and myelination of musculocutaneous nerve, prevented amyotrophy, facilitated neovascularization, and eventually resulted in better motor functional restoration of BPA rats. Based on those findings, we discovered that hydrogel-miR-615 could potentially protect motoneurons from BPA-induced stress by suppressing LINGO-1.

Our results demonstrated that LINGO-1 was highly expressed in the spinal cord of BPA-reimplantation rats. LINGO-1, a potent negative regulator of neuron survival, was highly expressed after various CNS injuries (Mi et al. 2008). Additionally, a previous study has shown that the expression of LINGO-1 is upregulated in the substantia nigra of Parkinson’s disease patients and in Parkinson’s disease mice models after neurotoxic lesions (Inoue et al. 2007). Our previous study also suggesting that the down regulation of LINGO-1 could promote motor functional recovery by protecting neurons from BPA-induced nerve injury in rats. Similarly, another study suggests that LINGO-1-RNAi-treated neural stem cell transplantation into rats improves functional recovery after complete spinal cord injury by promoting axonal regeneration and remyelination (Chen et al. 2016), which was consistent with our previous in vivo experimental results.

In addition, our recent study verified that LINGO-1 was a target gene of miR-615 using luciferase reporter assays and miR-615 overexpression could inhibit LINGO-1 expression, therefore contributing to neural stem cells differentiation in vitro and spinal cord recovery in vivo (Wu et al. 2020). Here, the current study suggests that miR-615 also could protect neuron against brachial plexus avulsion-induced motoneuron damage and loss. Generally, our current study is consistent with several researches supporting the neuroprotective effect of miR-615 in nervous system damage. Evidence from the brain ischemic injured animal models has shown that miR-615-3p might regulate conventional protein kinase C βII, γ, and novel PKC ε-interacting proteins, which were involved in hypoxic pre-conditioning-induced neuroprotection (Liu et al. 2012). Another research has also suggested that miR-615-5p acts as a protector against retinal neurodegeneration, which is inhibited by circular RNA-ZNF609, an endogenous miR-615 sponge. During retinal neurodegeneration, circular RNA-ZNF609 overexpression leads to the sink of miR-615 and removes the miR-615-mediated inhibitory role on METRN expression (Wang et al. 2018). Interestingly, miR-615-3p expression was increasing during the differentiation of human embryonic stem cells into neural precursors (Yan et al. 2018), suggesting that miR-615-3p may be involved in neural fate determination. Similar conclusions can also be drawn based on a present study which indicated that miR-615 could play a neuroprotective role after BPA-reimplantation by upregulating NeuN expression and blocking LINGO-1 expression.

Accumulating studies have described that the reactive astrocytes could undergo hypertrophy and aggravate neuronal damage after neurological injury through synthesizing and releasing pro-inflammatory cytokines, which can destruct local neurons (Farina et al. 2007). Therefore, inhibiting astrocyte activation could reduce neuronal injury and improve neurological functional recovery. In the current study, we found that miR-615 agomir with PF-127 hydrogel could significantly decrease astrocytes activation after BPA and provided an appropriated environment for motor nerve functional recovery in rats.

Increasing evidence indicates that neurogenesis and angiogenesis are strongly related (Ruan et al. 2015; Zhai and Feng 2019). Neurons and vascular endothelial cells are closely connected and are integrated within the neurovascular unit. Any injury to one component of this unit may destruct the system (Mabuchi et al. 2005). Importantly, most neural precursor cells prefer proliferating in angiogenic environment (Zhai and Feng, 2019). Meanwhile, endothelial cells could secrete various neuronal growth factors, such as BDNF, BMPs, and PEDF, which could promote neuronal precursor cell expansion (Butler et al. 2010). In the current study, to verify the effect of hydrogel-miR-615 compound on angiogenesis, we investigated neovascularization using immunofluorescence staining with CD31, a newborn microvessel marker. Our data suggest that miR-615 treatment may contribute to better neovascularization compared with PBS treatment. We also demonstrated the positive impact of hydrogel-miR-615 treatment on angiogenesis, neurogenesis, and motor functional recovery following BPA, but the mechanisms involved remain unidentified. Recent studies also suggest that miR-615-5p could protect human umbilical vein endothelial cells from hyperglycemia-induced apoptosis, inhibit pathological angiogenesis, and promote endothelial cell migration and tube formation of endothelial cells (Liu et al. 2017). However, another study indicates that miR-615-5p also has an anti-angiogenic effect by regulating the VEGF-AKT/eNOS pathway. In wounds of diabetic mice and skin of diabetics, the expression of miR-615-5p is increased, and aberrant expression of miR-615-5p significantly suppresses proliferation, migration, and tube formation of endothelial cells. Local administration of miR-615-5p inhibitor significantly increases angiogenesis and improves wound healing in diabetic mice (Icli et al. 2019). Therefore, neutralization of miR-615-5p may serve as a promising angiogenic therapy target which deserves us to further study.

In summary, hydrogel-miR-615 compound promoted neurogenesis at least in part by suppressing of LINGO-1, inhibited astrocyte activation, and promoted function recovery after BPA. These data indicate that miR-615 may serve as a potential therapeutic target for BPA treatment. However, the detailed mechanisms of miR-615 in neuroprotective effects in BPA rats still deserve further exploration.

## Data Availability

The materials used during the present study are available from the corresponding author on reasonable request.
